# HAX1 Promotes Hepatocellular Carcinoma Progression by Inhibiting Ferroptosis Through Modulation of Iron Homeostasis and the GSH/GPX4 Pathway

**DOI:** 10.3390/ijms27135935

**Published:** 2026-07-01

**Authors:** Yueyue Guo, Yuting Zhou, Jing Wu, Jizhe Zhou, Miaomiao Zhu, Delong Xie, Sangui Yi, Zongling Liu

**Affiliations:** 1 School of Basic Medical Sciences, Youjiang Medical University for Nationalities, Baise 533000, China; 2 Food Safety and Nutrition Experimental Teaching Demonstration Center, Department of Food Science and Engineering, Xinjiang Institute of Technology, Aksu 843000, China

**Keywords:** HAX1, ferroptosis, hepatocellular carcinoma, proliferation

## Abstract

Hepatocellular carcinoma (HCC) remains a malignancy with poor prognosis and limited therapeutic targets. Emerging evidence suggests a critical role for iron metabolism and ferroptosis in tumor progression. However, the involvement of hematopoietic lineage cell-specific protein 1 (HAX1) in HCC, particularly its regulatory role in ferroptosis, remains largely unknown. Here, we report that HAX1 is significantly upregulated in HCC tissues and correlates with advanced pathological stages and poor patient survival, suggesting its potential as an oncogene. Functionally, HAX1 overexpression promotes the proliferation and migration of HCC cells, while its knockdown inhibits these malignant phenotypes. Mechanistically, we demonstrate that HAX1 acts as a negative regulator of ferroptosis. Silencing HAX1 sensitizes HCC cells to the ferroptosis inducer IKE, leading to abnormal accumulation of intracellular ferrous iron (Fe^2+^) and increased lipid reactive oxygen species (ROS). Conversely, HAX1 overexpression suppresses iron overload and lipid peroxidation. Furthermore, we reveal that HAX1 maintains redox homeostasis by regulating the GSH/GPX4 antioxidant pathway. Knockdown of HAX1 depletes reduced glutathione (GSH), reduces glutathione peroxidase activity, and downregulates key ferroptosis defense proteins, including GPX4, FSP1, and SLC7A11. Our findings identify HAX1 as a critical promoter of HCC progression that functions by inhibiting ferroptosis through the modulation of iron homeostasis and the GSH/GPX4 pathway. Targeting the HAX1-mediated anti-ferroptotic mechanism may represent a promising therapeutic strategy for HCC treatment.

## 1. Introduction

Hepatocellular carcinoma (HCC) is recognized as the most common and highly malignant primary liver neoplasm, typically arising from hepatocytes in the context of chronic liver disease and cirrhosis [1]. Despite the prevailing treatment approach of surgical intervention coupled with systemic therapies (including targeted therapy and immunotherapy) for HCC, the 5-year survival rate for advanced-stage patients remains suboptimal, posing a significant risk to patients’ overall survival [2,3]. Therefore, it is urgent to explore novel targets for HCC therapy.

Ferroptosis, a novel iron-dependent regulated cell death (RCD) mechanism, is biochemically and morphologically distinct from classical cell death pathways like apoptosis and necrosis. This process is fundamentally driven by iron accumulation and lipid peroxidation [4,5]. A substantial body of research indicates that an imbalance in iron homeostasis and ferroptosis are closely associated with the malignant development of multiple tumor types. For instance, targeting macrophage xCT-mediated ferroptosis suppresses HCC progression and enhances anti-PD-1/PD-L1 immunotherapy [6]. However, cancer cells often exhibit multiple defense mechanisms that can block ferroptosis and facilitate tumor progression. For instance, PGAM1 acts as a critical suppressor of ferroptosis in hepatocellular carcinoma, thereby promoting tumor growth and impairing the efficacy of anti-PD-1 immunotherapy [7]. Moreover, the METTL16-SENP3-LTF pathway mediates ferroptosis resistance in cancer cells, further driving tumorigenesis and malignant progression in liver cancer [8]. In addition, the ATF4-SLC7A11/xCT axis serves as a key protective mechanism that inhibits stress-induced ferroptosis, which is closely associated with the development of hepatocellular carcinoma [9]. Therefore, identifying the defensive strategies used by cancer cells to evade ferroptosis is critical for improving the efficacy of cancer treatment.

HAX1 is a multifunctional mitochondrial and cytoplasmic protein that regulates cell survival, apoptosis, and migration and is closely associated with tumor progression and metastasis. A variety of tumors exhibit significant alterations in HAX1 expression levels. In non-small cell lung cancer, elevated HAX1 expression promotes malignant progression and correlates with poor clinical outcomes [10]. HAX1 overexpression suppresses autophagic flux and enhances chemoresistance in nasopharyngeal carcinoma by forming an IGF2BP1-HAX1 positive feedback loop [11]. The EIF3H-HAX1 axis stabilizes HAX1 protein and activates RAF-MEK-ERK signaling to drive tumorigenesis and metastasis in colorectal cancer [12]. In cervical cancer, HAX1 enhances cell survival, migration, and proliferation by maintaining mitochondrial integrity and reducing ROS accumulation, and these effects can be counteracted by wild-type p53 [13]. HAX1 is frequently upregulated in hepatocellular carcinoma (HCC) and promotes HCC cell migration, invasion, and metastasis by inducing epithelial–mesenchymal transition through the NF-κB signaling pathway [14,15]. Nevertheless, limited information is currently available regarding the involvement of HAX1 in ferroptosis.

In the current study, the association between HAX1 expression and prognosis in HCC was investigated. The impact of HAX1 on the proliferation of Huh7 and MHCC97H liver cancer cells was further evaluated using CCK-8 and colony formation assays. To elucidate the relationship between HAX1 and ferroptosis, HAX1 expression was modulated, followed by the assessment of ferroptosis-related proteins via Western blot, as well as the measurement of GSH, Fe^2+^, MDA levels, and GPX activity. Collectively, these findings provide a potential therapeutic target for the treatment of liver cancer.

## 2. Results

### 2.1. HAX1 Overexpressed in HCC and Correlates with a Poor Prognosis

**Figure 1:**
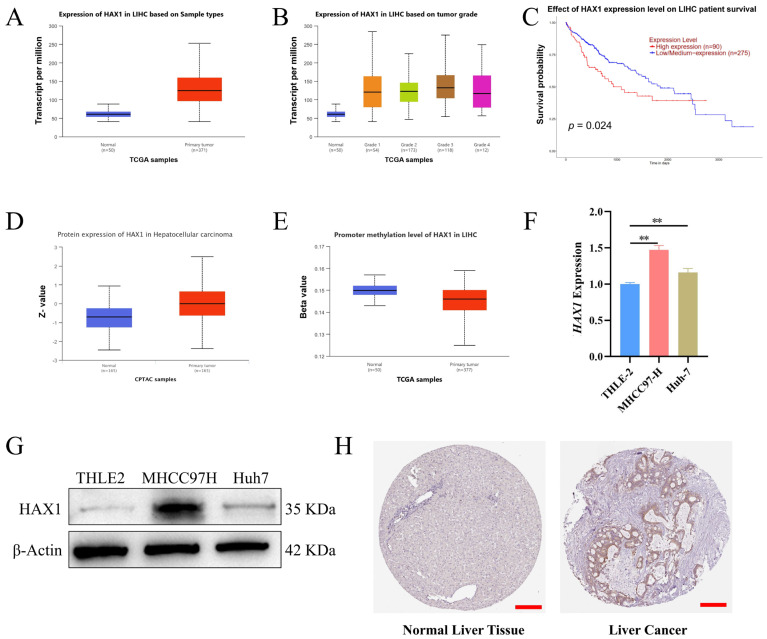
Elevated HAX1 expression in hepatocellular carcinoma (HCC): (**A**) HAX1 transcript levels in HCC versus normal tissues from The Cancer Genome Atlas (TCGA) database. (**B**) HAX1 expression across different pathological stages of HCC in TCGA cohort. (**C**) Correlation between HAX1 expression levels and overall survival of HCC patients in TCGA database. (**D**) HAX1 protein abundance in HCC tissues from the Clinical Proteomic Tumor Analysis Consortium (CPTAC) database. (**E**) HAX1 promoter methylation status in HCC tissues from TCGA database. (**F**,**G**) Original experimental data of HAX1 expression detected by RT-qPCR and Western blot in THLE-2, MHCC97H, and Huh7 cell lines. (**H**) Immunohistochemical staining of HAX1 in HCC and normal tissues from the Human Protein Atlas (HPA) database. Bars = 200 μm. Statistical analysis was conducted using a two-tailed Student’s *t*-test. Data are shown from three independent biological experiments, with two technical replicate wells per experiment. ** *p* < 0.01.

To elucidate the potential function of HAX1 in HCC, we conducted survival and differential expression analyses leveraging the UALCAN platform, which utilizes data from The Cancer Genome Atlas (TCGA) liver cancer cohort. The following observations were made: (1) Analysis revealed a significant upregulation of HAX1 expression in HCC tissues compared to adjacent normal controls (*p* < 0.01) (Figure 1A). (2) HAX1 levels were found to increase with disease progression, being significantly elevated in pathological stage 3 relative to stages 1 and 2 (Figure 1B). (3) High HAX1 expression was strongly associated with poor prognosis (*p* < 0.01) (Figure 1C). (4) A significant overexpression of HAX1 protein was observed in HCC tissues when compared to adjacent normal tissues (*p* < 0.01) (Figure 1D). (5) The promoter methylation level of HAX1 in HCC tissues was significantly lower than that in normal tissues (*p* < 0.01) (Figure 1E). Based on the comparison with normal cells, both HAX1 mRNA and protein levels were significantly increased in MHCC97H and Huh7 hepatocellular carcinoma cell lines relative to the immortalized normal liver epithelial cell line THLE2 (Figure 1F,G). Independent validation utilizing the Human Protein Atlas (HPA) dataset corroborated that elevated HAX1 expression is significantly associated with a detrimental prognosis in HCC patients (Figure 1H). Taken together, the results indicated that HAX1 may act as an oncogene in HCC.

### 2.2. HAX1 Increased the Proliferation and Migration of HCC Cells

**Figure 2:**
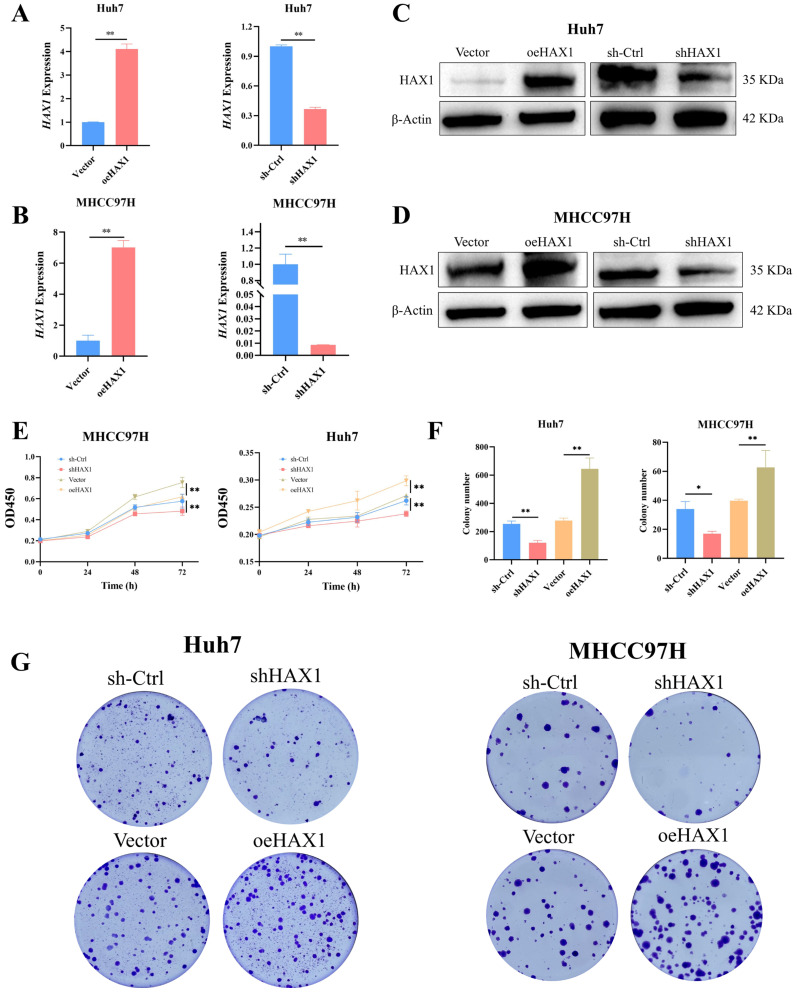
HAX1 drives proliferation and colony formation in HCC cell lines. Validation of HAX1 overexpression and knockdown efficiency at both mRNA (**A**,**B**) and protein (**C**,**D**) levels in MHCC97H and Huh7 cells. (**E**) CCK-8 assays assessing the impact of HAX1 modulation on cell proliferation. (**F**,**G**) Representative images and quantification of colony formation assays showing the effect of HAX1 on clonogenic survival. Statistical analysis was conducted using a two-tailed Student’s *t*-test. Data are shown from three independent biological experiments, with two technical replicate wells per experiment. * *p* < 0.05. ** *p* < 0.01.

To further elucidate the functional role of HAX1 in modulating the malignant progression of Huh7 and MHCC97H cells, stable cell lines with ectopic HAX1 overexpression and downregulation were successfully established. The efficiency of HAX1 modulation was rigorously confirmed at both the transcriptional and translational levels by qPCR (Figure 2A,B) and Western blot (Figure 2C,D) analyses, respectively. Results from the CCK-8 proliferation assay demonstrated that enforced HAX1 expression significantly increased the proliferative capacity of Huh7 and MHCC97H cells, whereas HAX1 knockdown markedly suppressed cell proliferation (Figure 2E). Consistently, the colony formation assay revealed that HAX1 overexpression markedly increased the clonogenic potential, while silencing HAX1 led to a significant reduction in colony numbers in both cell lines (Figure 2F,G). Furthermore, the wound-healing assay indicated that upregulation of HAX1 accelerated the migratory ability of Huh7 and MHCC97H cells, in contrast to the impaired migration observed in the knockdown groups (Figure S1). Collectively, these functional experiments demonstrate that HAX1 acts as an oncogene to promote the malignant progression of Huh7 and MHCC97H cells.

### 2.3. HAX1 Knockdown Sensitizes HCC Cells to IKE-Induced Ferroptosis and Triggers Iron Overload

**Figure 3:**
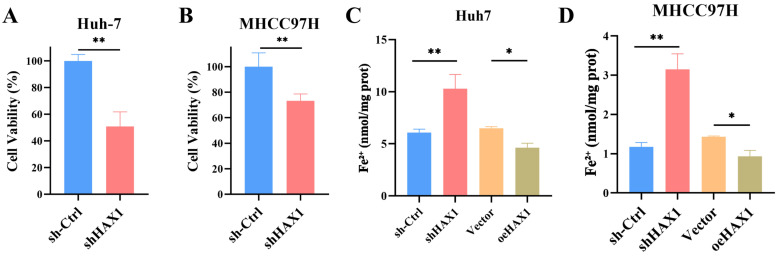
HAX1 silencing elevates the level of intracellular labile ferrous iron: (**A**,**B**) The depletion of HAX1 resulted in an increased sensitivity of Huh7 and MHCC97H cells to imidazole ketone erastin (IKE) at a concentration of 40 μM. (**C**,**D**) Intracellular ferrous iron (Fe^2+^) steady-state levels were detected in MHCC97H and Huh7 cells with HAX1 knockdown or HAX1 overexpression. Statistical analysis was conducted using a two-tailed Student’s *t*-test. Data are shown from three independent biological experiments, with two technical replicate wells per experiment. * *p* < 0.05. ** *p* < 0.01.

To elucidate the potential regulatory role of HAX1 in ferroptosis within HCC cells, we employed the specific ferroptosis inducer, imidazole ketone erastin (IKE), at a concentration of 40 μM, to investigate the functional involvement of HAX1. Our results demonstrated a significant negative correlation between HAX1 expression and the sensitivity of HCC cells to ferroptosis. Specifically, the depletion of HAX1 rendered Huh7 and MHCC97H cells markedly more sensitive to IKE-induced cytotoxicity, suggesting that the loss of HAX1 compromises cellular defense mechanisms (Figure 3A,B). Further mechanistic exploration revealed alterations in intracellular iron metabolism: overexpression of HAX1 significantly reduced intracellular ferrous iron (Fe^2+^) steady-state levels (Figure 3C,D). Conversely, knockdown of HAX1 induced the opposite effect, leading to abnormal accumulation of intracellular Fe^2+^. These findings suggest that HAX1 plays a pivotal role in suppressing ferroptosis and maintaining HCC cell survival, potentially by modulating intracellular iron homeostasis.

### 2.4. Silencing HAX1 Leads to the Accumulation of Lipid ROS in HCC Cells

**Figure 4:**
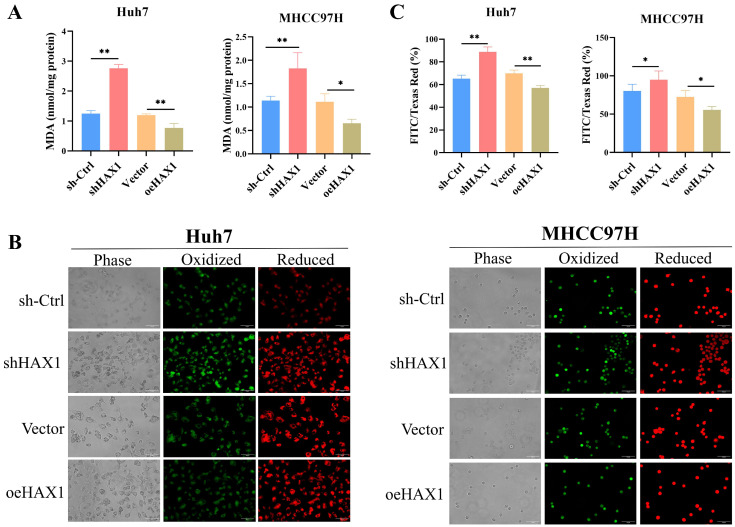
HAX1 knockdown leads to the accumulation of lipid peroxides in Huh7 and MHCC97H cells: (**A**) Malondialdehyde (MDA) levels were detected in MHCC97H and Huh7 cells with HAX1 knockdown or HAX1 overexpression. (**B**,**C**) Detection of lipid peroxidation in HAX1 knockdown and overexpression cells using the C11-BODIPY fluorescent probe. Scale bar, 100 µm. Statistical analysis was conducted using a two-tailed Student’s *t*-test. Data are shown from three independent biological experiments, with two technical replicate wells per experiment. * *p* < 0.05. ** *p* < 0.01.

To further elucidate the biological roles of HAX1 in lipid ROS accumulation, lipid peroxidation was assessed using MDA assays and the BODIPY 581/591 C11 probe. The results demonstrated that HAX1 overexpression resulted in decreased MDA levels, whereas its inhibition led to increased MDA levels in MHCC97H and Huh7 cells (Figure 4A). Furthermore, analysis using the fluorescent probe BODIPY 581/591 C11 revealed that HAX1 knockdown significantly exacerbated lipid peroxidation, while overexpression ameliorated this effect in both cell lines (Figure 4B,C). Collectively, these findings indicate that HAX1 plays a critical role in regulating lipid ROS accumulation.

### 2.5. HAX1 Regulates the GSH/GPX4 Antioxidant Pathway to Inhibit Ferroptosis

**Figure 5:**
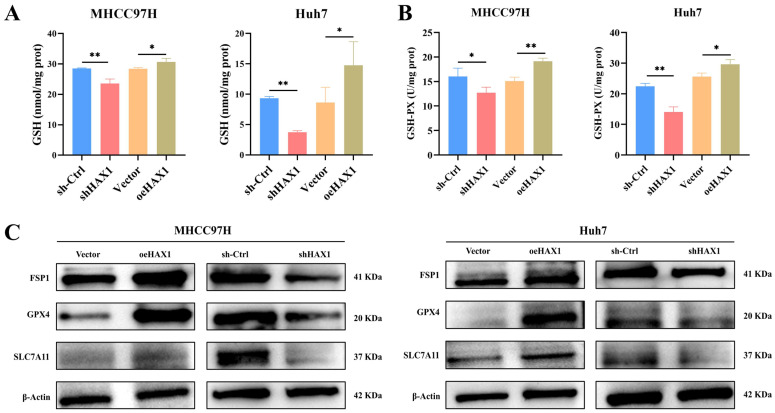
HAX1 is associated with the GSH/GPX4 pathway during ferroptosis suppression: (**A**) Measurement of intracellular reduced glutathione (GSH) levels following HAX1 overexpression or knockdown in Huh7 and MHCC97H cells. (**B**) Measurement of glutathione peroxidase (GSH-PX) activity following HAX1 overexpression or knockdown in Huh7 and MHCC97H cells. Data are shown from three independent biological experiments, with two technical replicate wells per experiment. (**C**) Western blot analysis showing the expression levels of key ferroptosis-related proteins (GPX4, FSP1, and SLC7A11) in Huh7 and MHCC97H cells upon HAX1 overexpression or knockdown. * *p* < 0.05. ** *p* < 0.01.

To investigate the role of HAX1 in regulating the GSH/GPX4 antioxidant pathway, we measured the intracellular levels of glutathione (GSH) and glutathione peroxidase (GSH-PX) following modulation of HAX1 expression. The results demonstrate a strong positive correlation between HAX1 expression and the activity of this antioxidant pathway. Specifically, in HAX1-knockdown cell models, cellular antioxidant capacity was significantly impaired. Compared to the control group, the levels of reduced glutathione (GSH) were markedly decreased (Figure 5A), and the activity/expression of glutathione peroxidase (GSH-PX) was also significantly reduced (Figure 5B). Consistent with these findings, Western blot analysis of key ferroptosis-related proteins revealed that knockdown of HAX1 led to a significant downregulation of GPX4, FSP1, and SLC7A11 in both Huh7 and MHCC97H cell lines. Conversely, overexpression of HAX1 resulted in a notable upregulation of these proteins (Figure 5C and Figure S2). These findings indicate that HAX1 positively regulates key molecules within the GSH/GPX4 antioxidant pathway. By maintaining GSH homeostasis and the expression of ferroptosis defense systems, HAX1 plays a crucial role in inhibiting ferroptosis.

## 3. Discussion

Ferroptosis, an iron-dependent form of regulated cell death (RCD) governed by GPX4, is fundamentally driven by the catastrophic accumulation of lipid peroxides resulting from reactive oxygen species (ROS) generation and iron availability [16]. Accumulating evidence underscores the pivotal role of ferroptosis in the progression of hepatocellular carcinoma (HCC). Notably, several therapeutic agents have demonstrated anti-tumor efficacy and synergistic potential with standard chemotherapeutics by targeting ferroptosis-related regulatory molecules in HCC [7,17]. Consequently, the identification of these critical regulators offers a promising avenue for enhancing HCC therapeutic strategies.

The role of HAX1 in cell apoptosis has been extensively investigated in multiple studies. HAX1 acts as a critical anti-apoptotic regulator by interacting with mitochondrial proteins such as HtrA2/Omi to prevent cytochrome c release and caspase activation, thereby maintaining mitochondrial membrane potential and cellular survival [18]. In the context of spinal cord injury, HAX1 expression is significantly upregulated and plays a protective role by inhibiting neuronal apoptosis, while its silencing promotes cell death [19]. Furthermore, HAX1 regulates neutrophil apoptosis by maintaining mitochondrial integrity, as its deletion leads to mitochondrial dysfunction and the activation of the intrinsic apoptotic pathway involving BCL-2 family members and Caspase-9 [13]. Our investigation provides the first evidence linking HAX1 to the regulation of ferroptosis. We observed that HAX1 is upregulated in HCC tissues, and this overexpression is significantly associated with an unfavorable clinical prognosis. Mechanistically, silencing HAX1 was shown to suppress HCC cell proliferation by triggering ferroptotic cell death. Collectively, these results position HAX1 as a promising prognostic biomarker and a novel therapeutic target for the treatment of HCC.

The fundamental cause of ferroptosis is the lethal accumulation of intracellular ROS, particularly lipid peroxides (lipid ROS). HAX1 directly interacts with the non-receptor tyrosine kinase c-Abl. Under conditions of oxidative stress, HAX1 facilitates the activation of c-Abl. Activated c-Abl modulates the degradation of H_2_O_2_, which may prevent lipid peroxidation levels from reaching the threshold required to trigger ferroptosis [20]. Corroborating this mechanism, our study demonstrated that HAX1 overexpression led to a significant reduction in MDA levels. Mitochondria serve as the primary site for ferroptosis, characterized morphologically by mitochondrial shrinkage, increased membrane density, and reduced cristae. HAX1 interacts with the mitochondrial matrix protein Cyclophilin D (Cyp-D) and negatively regulates its expression or function. Cyp-D is a key regulator of mPTP opening. By inhibiting Cyp-D, HAX1 prevents the excessive opening of the mPTP [21]. HAX1 may block the cascade of mitochondrial damage during the execution phase of ferroptosis by maintaining mitochondrial membrane integrity. HAX1 binds to InsP3R1, reducing calcium release from the endoplasmic reticulum and preventing mitochondrial calcium overload [22]. Calcium overload may activate mitochondrial MCU and NOX4, promoting Fe^2+^ release and ROS generation, thereby blocking the initiation of ferroptosis. Consistent with this view, in the present study, HAX1 knockdown promoted intracellular iron concentration. Collectively, HAX1 may suppress ferroptosis by reducing lipid peroxide accumulation, maintaining mitochondrial membrane integrity, and inhibiting mitochondrial calcium overload and intracellular iron elevation.

The present study preliminarily analyzed the relationship between HAX1 and ferroptosis in HCC. Nevertheless, there are still several limitations in the present study. This research only focused on phenotypic validation and macroscopic functional observation and did not further explore the specific molecular interaction and downstream signaling regulatory mechanism of HAX1 participating in ferroptosis. The exact molecular mechanism by which HAX1 modulates ferroptosis in hepatocellular carcinoma remains unclear. In future research, we will further investigate the in-depth molecular mechanism underlying the correlation between HAX1 and ferroptosis.

## 4. Materials and Methods

### 4.1. Cell Line and Culture

The human hepatocellular carcinoma (HCC) cell lines MHCC97H (Cat. No. C5108) and Huh7 (Cat. No. C5176), together with the immortalized normal human hepatic cell line THLE-2 (Cat. No. C5664), were purchased from Baidi Biotech Ltd. (Shanghai, China). The cells were cultured in DMEM medium (Servicebio, Wuhan, China, Cat. No. G4511) supplemented with 10% fetal bovine serum (Biosharp, Beijing, China, Cat. No. BL205A) and 1% penicillin–streptomycin solution (Servicebio, Cat. No. G4003). All cells were maintained in a humidified incubator at 37 °C in an atmosphere of 5% CO_2_.

### 4.2. Plasmid Constructions and Transfection

To generate stable cell lines, the PiggyBac transposon system [23], including plasmids of PiggyBac Dual Promoter (PB) and Super PiggyBac Transposase (SPB), was employed to establish MHCC97H and Huh7 cells with either HAX1 overexpression or knockdown. The coding sequence (ORF) for HAX1 and specific shRNA sequences targeting HAX1 were synthesized by Binhui Biopharmaceutical Co., Ltd. (Wuhan, China) and subsequently inserted into the PB-flag and PB-U6P vectors, respectively. The resulting constructs were named PB-HAX1-flag (overexpression) and PB-U6P-ShHAX1 (knockdown). For the transfection procedure, MHCC97H and Huh7 cells were plated in 6-well dishes at a density of 5 × 10^5^ cells/well. Once attached, the cells were co-transfected with 500 ng of the respective recombinant plasmid and 500 ng of SPB plasmid using Lipo8000 (Beyotime, Shanghai, China, Cat. No. C0533), strictly following the manufacturer’s guidelines. Control groups were transfected with empty PB-flag or PB-U6P vectors alongside the SPB plasmid. Selection for stable integrants was initiated 48 h post-transfection by culturing the cells in medium supplemented with 2 µg/mL puromycin (Beyotime, Cat. No. ST551).

### 4.3. Cell Counting Kit-8 Assay

To evaluate cell proliferation, MHCC97H and Huh7 cells were plated in 96-well plates at a density of 2000 cells per well and cultured for 72 h at 37 °C in a 5% CO_2_ atmosphere. Following the incubation period, 10 µL of CCK-8 solution (UElandy, Suzhou, China, Cat. No. C6005M) was added to each well. The plates were then returned to the incubator for an additional 2 h [24]. Finally, cell viability was assessed by measuring the absorbance at 450 nm using a microplate reader. Data are shown from three independent biological experiments, with two technical replicate wells per experiment.

### 4.4. Colony Formation Assay

MHCC97H and Huh7 cells were seeded into 6-well plates at a density of 5000 cells per well. The cells were cultured for 7 d at 37 °C with 5% CO_2_ to allow for colony development, with the culture medium being replaced every 2 d. Upon completion of the incubation period, the colonies were fixed using 4% paraformaldehyde (Servicebio, Cat. No. G1101) for 30 min at room temperature. Following fixation, the cells were stained with crystal violet solution (Beyotime, Cat. No. C0121) for 10 min at room temperature [24]. The condition of cell colonies across all wells was imaged and quantified. Data are shown from three independent biological experiments, with two technical replicate wells per experiment.

### 4.5. Wound-Healing Assay

Cells were seeded into 6-well plates at a density of 1 × 10^6^ cells per well and cultured overnight until they reached full confluence. A uniform wound was generated across the cell monolayer using a 10 µL pipette tip. Following the scratch, cells were incubated at 37 °C with 5% CO_2_. Images of the wound area were captured at 0, 24, and 48 h using a light microscope [24]. The remaining scratch areas were measured with ImageJ v1.54 software to calculate the wound closure rate, which served as an indicator of cell migration ability. Data are shown from three independent biological experiments, with two technical replicate wells per experiment.

### 4.6. Reverse-Transcription Quantitative PCR (RT-qPCR)

Total RNA was isolated using the HiPure Total RNA Mini Kit (Magen, Guangzhou, China, Cat. No. R4111-02), and the concentration was quantified using a TGem Plus full-wavelength spectrophotometer (Tiangen Biochemical Technology Co., Ltd., Beijing, China). Subsequently, cDNA synthesis was performed using the BeyoRT™ III cDNA first-strand synthesis premix (with gDNA EZeraser) (Beyotime, Cat. No. D7185S). Quantitative real-time PCR (qPCR) was conducted using BeyoFast™ SYBR Green qPCR Mix (2X) (Beyotime, Cat. No. D7260). The relative expression levels were determined via the 2^−ΔΔCT^ method, with GAPDH serving as an internal reference gene [25]. The specific primer sequences utilized in this study are listed in Table S1. Data are shown from three independent biological experiments, with two technical replicate wells per experiment.

### 4.7. Western Blot

Cells were harvested and lysed on ice for 10 min using RIPA buffer (Beyotime, Cat. No. P0013B) supplemented with PMSF. The lysates were then centrifuged at 12,000 rpm for 15 min at 4 °C to remove cellular debris. Protein concentration was quantified using a BCA Kit (Beyotime, Cat. No. P0011). Equal amounts of protein (50 μg) were separated by 10% SDS-PAGE and transferred to membranes for immunoblotting [24]. The membranes were probed with the following primary antibodies: HAX1 (1:1000; Abclonal, Wuhan, China, Cat. No. A5551), GPX4 (1:7500; Proteintech, Wuhan, China, Cat. No. 82822-2-RR), SLC7A11 (1:4500; Proteintech, Cat. No. 82115-2-RR), FSP1 (1:4000; Proteintech, Cat. No. 20886-1-AP), β-Actin (1:50,000; Proteintech, Cat. No. 10052031), and GAPDH (1:250,000; Proteintech, Cat. No. 60004-1-1g).

### 4.8. Assessment of Glutathione Homeostasis

As for glutathione (GSH) detection, cells were resuspended with 500 μL of PBS added per approximately 1 × 10^6^ cells. Following homogenization, the mixture was centrifuged at 10,000× *g* for 10–15 min at 4 °C. The final supernatant was harvested for subsequent reduced GSH measurement. Intracellular GSH levels in MHCC97H and Huh7 cells were determined using a Glutathione Assay Kit (Servicebio, Cat. No. G4305) following the manufacturer’s protocol. As for glutathione peroxidase (GPX), an aliquot of 200 μL PBS was added per approximately 1 × 10^6^ cells for homogenization and lysis. The homogenate was centrifuged at 10,000× *g* for 10–15 min at 4 °C, and the supernatant was collected on ice for subsequent detection. The total activity of GPX was assessed using a specific detection kit (Servicebio, Cat. No. G4310) following the manufacturer’s protocol. Data are shown from three independent biological experiments, with two technical replicate wells per experiment.

### 4.9. Measurement of Lipid Peroxides

As for malondialdehyde (MDA) measurement, cells were resuspended in IP lysis buffer, with 200 μL of reagent added per approximately 1 × 10^7^ cells, followed by sonication or lysis on ice. The lysate was centrifuged at 10,000× *g* for 10–15 min at 4 °C, and the supernatant was collected for subsequent MDA measurement. MDA was evaluated by the Malondialdehyde (MDA) Assay Kit (Servicebio, Cat. No. G4300). As for the BDPY 581/591 C11 assay, an aliquot of 1 mL BODIPY 581/591 C11 working staining solution (Beyotime, Cat. No. S0043S) was added, and cells were incubated at 37 °C in an incubator for 20 min. Following incubation, the supernatant was aspirated, the cells were washed twice with PBS, 2 mL of PBS was added, and samples were visualized under a fluorescence microscope. The fluorescence intensity was quantitatively analyzed using a microplate reader (Tecan, Maennedorf, Switzerland, Spark). Data are shown from three independent biological experiments, with two technical replicate wells per experiment.

### 4.10. Intracellular Iron Levels

An aliquot of 500 μL extraction buffer was added per ~1 × 10^6^ cells for homogenization. The homogenate was centrifuged at 10,000× *g* for 10–15 min at 4 °C, and the supernatant was collected for subsequent assays. The BCA method could be used to determine total protein concentration. Iron content was quantified using an Iron Assay Kit (Servicebio, Cat. No. G4323) following the manufacturer’s protocol. Data are shown from three independent biological experiments, with two technical replicate wells per experiment.

### 4.11. Statistical Analysis

All statistical analyses were carried out with SPSS 20.0, and quantitative data are presented as mean ± standard deviation (SD). For two-group comparisons, a two-sided unpaired Student’s *t*-test was adopted. For multi-group comparisons, one-way analysis of variance (ANOVA) followed by post hoc Tukey’s multiple-comparison test was used, with Tukey correction applied to control type I error for multiple comparisons. Data presented herein were generated across three independent biological experiments, each containing two technical replicate wells. Two-tailed *p* values were reported, and *p* < 0.05 was defined as statistically significant.

## 5. Conclusions

In summary, HAX1 is aberrantly overexpressed in HCC tissues and cell lines, and its high expression correlates with poor prognosis, functioning as an oncogene. HAX1 promotes malignant phenotypes in HCC cell lines. Our data suggest that this oncogenic role is likely associated with its capacity to limit ferroptosis via the maintenance of iron homeostasis and the GSH/GPX4 axis. Our findings identify HAX1 as a key regulator of HCC progression and ferroptosis, offering a potential target for ferroptosis-based HCC therapy.

## Data Availability

The original contributions presented in this study are included in the article/Supplementary Materials. Further inquiries can be directed to the corresponding authors.

## Supplementary Materials

The following supporting information can be downloaded at: https://www.mdpi.com/article/10.3390/ijms27135935/s1.
