# Supplementary material for: HAX1 Promotes Hepatocellular Carcinoma Progression by Inhibiting Ferroptosis Through Modulation of Iron Homeostasis and the GSH/GPX4 Pathway
Source: Int J Mol Sci. 2026 Jul 1;27(13):5935. doi: 10.3390/ijms27135935 (PMC13361179; doi:10.3390/ijms27135935)
Supplement: Supplementary file 1 [file ijms-27-05935-s001.zip › ijms-4367956-supplementary.pdf]

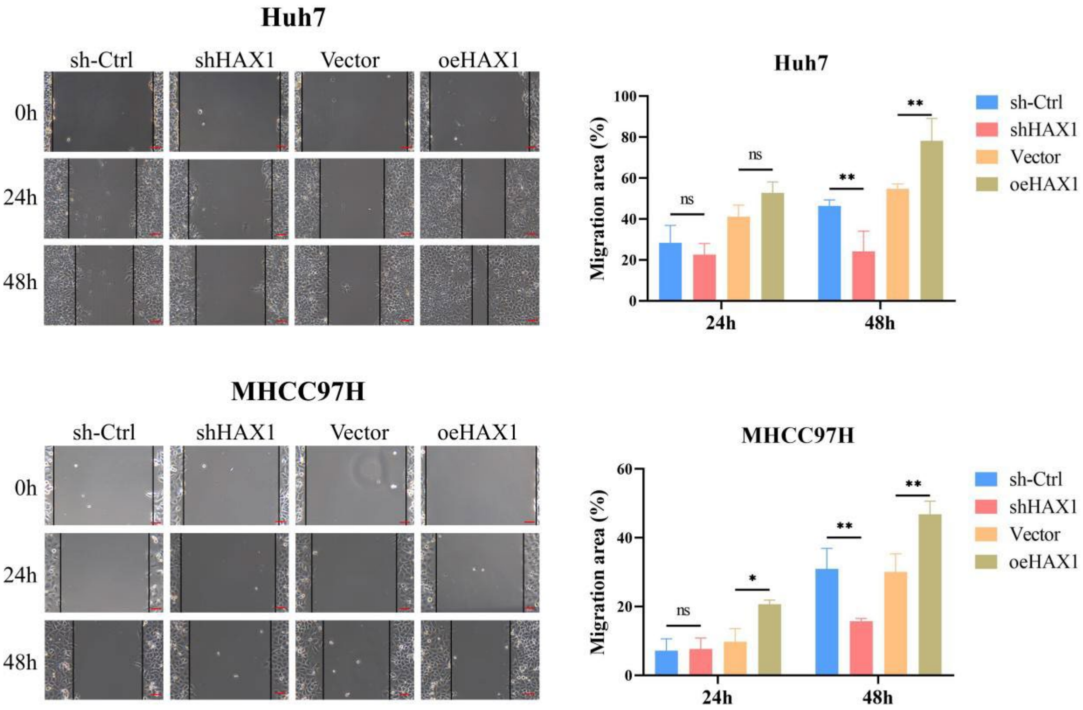

Figure S1. HAX1 promotes the migration of Huh7 and MHCC97H cells. Scale bar, 100 μm. * $p < 0.05$, ** $p < 0.01$, ns, not significant.

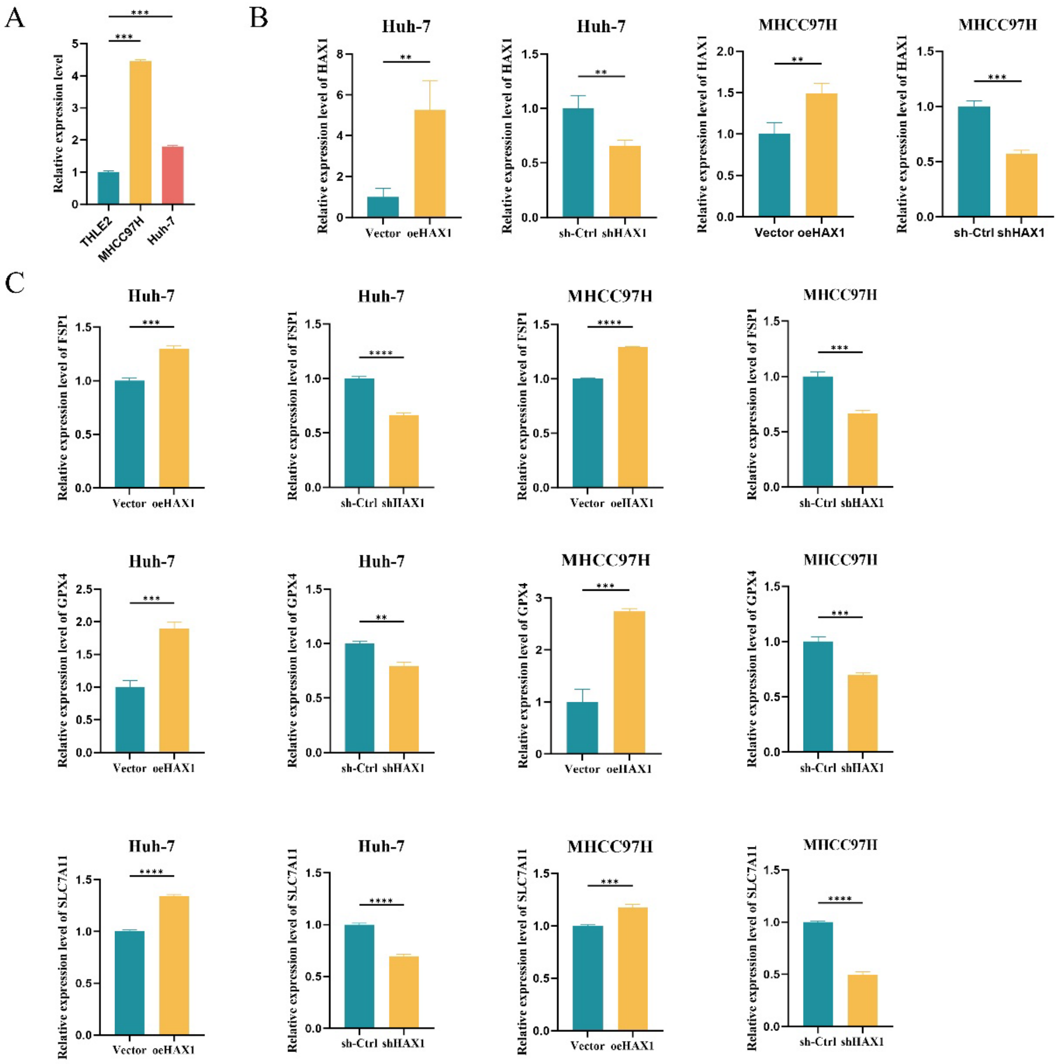

Figure S2. Quantitative analysis of western blot bands from Figure 1 (A), Figure 2 (B) and Figure 5 (C). ** $p < 0.01$, *** $p < 0.001$, **** $p < 0.0001$.

Table S1. Primers used in this study.

Primer Name	Sequence (5′-3′)
qPCR-HAX1-F	CAGGAGGAGGGATACGTTTCC
qPCR-HAX1-R	CCCATATCGCTGAAGATGCTATT
qPCR-GAPDH-F	GTCTCCTCTGACTTCAACAGCG
qPCR-GAPDH-R	ACCACCCTGTTGCTGTAGCCAA
